# Adaptation of *Anaplasma phagocytophilum* to the tick vector is controlled by the transcriptional regulator Tr1

**DOI:** 10.1128/msphere.00872-25

**Published:** 2026-06-15

**Authors:** EricaRose Warwick, Rachel Burt, Jeffrey T. Badigian, Daniel Howell, Kyle T. Swallow, Chloe Leach, Azeza M. Falghoush, Dana K. Shaw, Ian T. Cadby, Jason M. Park

**Affiliations:** 1 Department of Veterinary Microbiology and Pathology, Washington State University 312980, Pullman, Washington, USA; 2 Bristol Veterinary School, University of Bristol 1980, Bristol, United Kingdom; 3 College of Sciences, Sirte University 267350, Sirte, Libya; 4 College of Medical Technology, Aljufra University, Aljufra, Libya; Virginia Tech, Blacksburg, Virginia, USA

**Keywords:** *Anaplasma*, *Anaplasma phagocytophilum*, *Ixodes*, tick-borne disease, transcription factor, transcriptional regulation, type IV secretion system, T4SS effectors, bacterial effectors, membrane proteins

## Abstract

Rickettsial pathogens are strictly dependent on the cellular biology of their hosts for survival and replication. Predominantly transmitted by blood-feeding arthropods, these vector-borne pathogens are forced to adapt between the disparate environments of their mammalian host and arthropod vector. To achieve this, the Rickettsial bacteria *Anaplasma phagocytophilum* undergo extensive transcriptional reprogramming, with over 41% of its genes differentially transcribed between mammals and *Ixodes scapularis* ticks. How the bacterium orchestrates this dramatic transcriptional reprogramming is not understood. The gene *tr1* encodes a Helix-Turn-Helix DNA-binding protein that is exclusively expressed during tick infection. Herein, we show that *tr1* is essential for *A. phagocytophilum* survival in ticks and regulates the transcription of other genes necessary to adapt to the arthropod vector. We demonstrate that Tr1 is a DNA-binding protein that recognizes promoters of tick-specific genes in *A. phagocytophilum*, including secreted effector *ateA*, alternate components of the type IV secretion system (T4SS), and membrane proteins. Our findings demonstrate that Tr1 is a master regulator of genes critical for *A. phagocytophilum* adaptation to the tick.

**IMPORTANCE:**

Tick-borne pathogens are a persistent threat to human and animal health worldwide. These pathogens must be capable of surviving in both the arthropod vector and the mammalian hosts to successfully complete their lifecycle. To achieve this, these pathogens reciprocally regulate genes that are specific for either mammalian or tick infection. The mechanism orchestrating this switch remains undefined. In this study, we identify a transcriptional regulator controlling how the tick-borne agent for granulocytic anaplasmosis, *Anaplasma phagocytophilum*, adapts to life in the tick. Disabling this transcriptional switch and the genes it controls renders the bacteria unable to survive in the arthropod vector. Understanding how this central regulator and the genes under its control impact tick infection could lead to interventions that disrupt the cycle of transmission, thereby preventing disease.

## INTRODUCTION

Rickettsial pathogens are strictly dependent on the cellular biology of their hosts for survival and replication, which is reflected by their small genomes and reduced metabolic capacities. Instead, these bacteria have evolved factors to mediate host-pathogen interactions and manipulate the intracellular environment (1–4). These include specialized surface proteins mediating contact and uptake into cells (2, 3, 5–9) and effector molecules injected through secretion systems to redirect host cell pathways (10–23). The large majority of rickettsial host-pathogen interactions are described in the context of mammalian infection (17, 20–22, 24, 25). However, since rickettsial bacteria are predominantly transmitted by blood-feeding arthropods, these findings only explore half of the pathogen lifecycle (26). Mammals and arthropods are separated by over 680 million years of evolution and, as such, present distinct environments that rickettsial pathogens must adapt to survive intracellularly (27).

The most common rickettsial pathogen in the United States, *Anaplasma phagocytophilum*, is transmitted to humans, domestic animals, and wildlife by the ticks *Ixodes scapularis* and *Ixodes pacificus* (28). While in the mammalian host, *A. phagocytophilum* infects and replicates within circulating neutrophils. In the tick, the bacteria initially infect the digestive tract and then migrate to the salivary glands, where they will survive through the molt (29, 30). In response to the disparate biology between the mammalian host and tick vector (31), *A. phagocytophilum* undergoes extensive transcriptional reprogramming. Transcriptomic studies found that over 41% of *A. phagocytophilum* genes are differentially transcribed when comparing infected human monocytes and tick cells (32, 33). Indeed, Himar1 transposon disruption of host- or vector-specific *A. phagocytophilum* genes reduces bacterial survival in the respective human or tick cell cultures (10, 34–37). Together, this suggests that transcriptional reprogramming of *A. phagocytophilum* is necessary for adaptation between the host and vector environments.

While both transcriptomics and proteomics have shown *A. phagocytophilum* and other rickettsial pathogens undergo extensive retooling during tick infections (5, 32, 33, 38–42), the regulatory mechanisms that control these dramatic shifts are not known. One gene, *tr1*, encodes a putative Helix-Turn-Helix DNA-binding protein (Tr1). *tr1* was first noticed due to its proximity to outer membrane protein (OMP) genes *omp_1X*, *omp_1N,* and the *msp2* expression site (43, 44), but its impact on this neighboring operon is not defined. Strikingly, of all *A. phagocytophilum* genes, *tr1* has the highest transcriptional specificity for tick cell infection (32, 33). Herein, we demonstrate that *tr1* is essential for *A. phagocytophilum* survival in tick cells, colonization of ticks *in vivo*, and expression of many genes necessary for adaptation to the tick. Further, we demonstrate that Tr1 is a DNA-binding protein and recognizes promoters of tick-specific genes in *A. phagocytophilum*, including secreted effector *ateA*, alternate components of the type IV secretion system (T4SS), and membrane proteins. From this work, we have identified Tr1 as a master regulator of genes critical for *A. phagocytophilum* adaptation to the tick.

## RESULTS

### Transcription of tr1 is specific to tick cell infection

**Fig 1:**
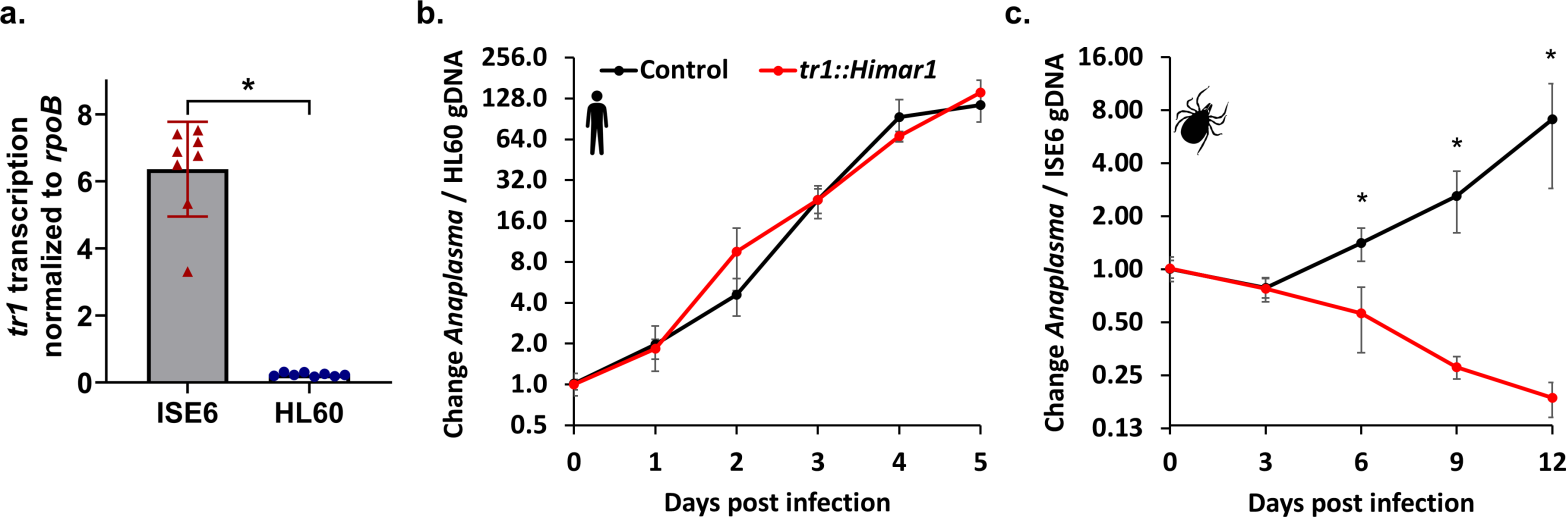
*tr1* transcription is specific to tick cell infection and necessary for *A. phagocytophilum* survival in tick cells. (**a**) *tr1* transcription normalized to housekeeping gene *rpoB* during *A. phagocytophilum* culture in ISE6 tick cells and human HL60 cells. Each bar indicates the mean with ± SD of four replicate infections, each measured in two technical replicates shown as points. (**b and c**) Growth of *A. phagocytophilum tr1*::Himar1 or control strain in cell culture infections of (**b**) human HL60 cells and (**c**) tick ISE6 cells. *A. phagocytophilum* burden measured by bacterial gDNA relative to eukaryotic host gDNA via qPCR. Data displayed as mean with ± SD of three biological infection replicates within an experiment. Each replicate was measured by qPCR in duplicate. Graphed are data from one experiment and are representative of three experimental replicates. *P < 0.05 (Mann-Whitney *t*-test).

*A. phagocytophilum* HGE1 *tr1* transcription was quantified and compared between bacteria cultured in the human monocyte-like HL60 cell line and embryonic tick ISE6 cells. Consistent with previous tiling array reports, *tr1* transcription was >26-fold higher during growth in tick ISE6 cells over the human HL60 cell line (Fig. 1a), validating that *tr1* expression is highly specific to growth in tick cells (32, 33).

### tr1 is necessary for *A. phagocytophilum* survival in tick cells

The specificity of *tr1* transcription to ISE6 cell culture prompted us to test if *tr1* is necessary for *A. phagocytophilum* adaptation to ticks. To examine this possibility, we obtained a transposon mutant strain *tr1*::Himar1 from the previously published mutant collection (34). An established control strain with the Himar1 transposon inserted in an intergenic non-coding region was used for comparison. This control strain is phenotypically comparable to wild type in both mammalian and tick infection models (10, 34, 36, 37). *tr1::Himar1* and the control *A. phagocytophilum* strain were purified from HL60 cell culture and used to infect either ISE6 or HL60 cells. Bacterial burdens were compared over time. During HL60 infection, the control and *tr1*::Himar1 strains grew equivalently (Fig. 1b). However, during tick cell infection, *tr1*::Himar1 steadily declined (Fig. 1c), indicating that it is required for *A. phagocytophilum* adaptation to tick cells.

### *A. phagocytophilum* requires tr1 to colonize ticks *in vivo*

**Fig 2:**
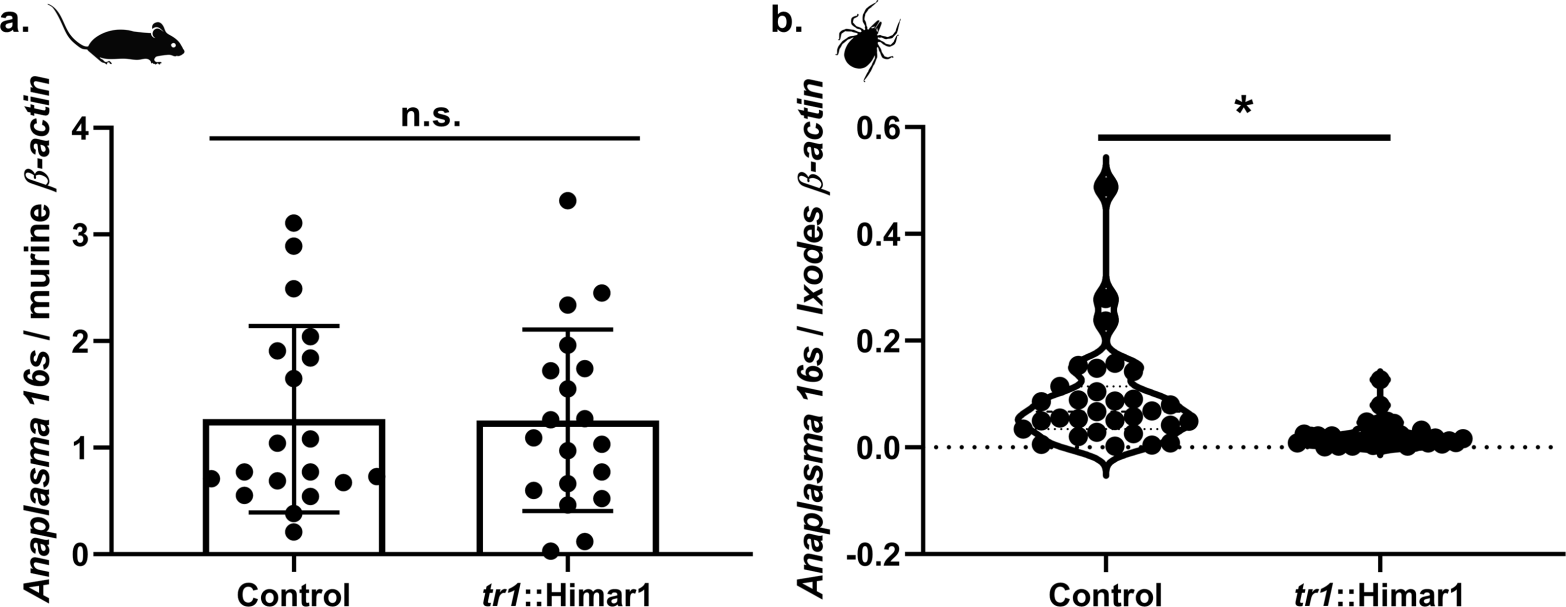
*tr1* is despicable during murine infection but essential for survival in ticks. (**a**) Mouse blood *Anaplasma* burden 7 days post-intraperitoneal inoculation with 1 × 10^8^ *A. phagocytophilum tr1*::Himar1 or control::Himar1 strains. Blood processed for gDNA and bacterial burden was measured by qPCR of *A. phagocytophilum* 16S rDNA versus mouse actin by ΔΔCt. Each strain was tested in 20 mice (1/2 male, 1/2 female). Each sample was tested in technical duplicate reaction. (**b**) Two burden-matched infected mouse pairs were used for *Ixodes scapularis* larvae infestation. Ticks were allowed to feed to repletion and detach. Whole replete *I. scapularis* larvae were processed for RNA. *A. phagocytophilum* bacterial loads were measured via qRT-PCR of *A. phagocytophilum* 16S rRNA levels versus mouse actin transcripts. Data includes ticks from two burden matched mouse pairs. From each mouse, 10–20 individual ticks were collected as biological replicates, and each qRT-PCR was performed in duplicate. *P < 0.005 (Welch’s *t*-test).

The *in vitro tr1*::Himar1 phenotype led us to ask if *tr1* is similarly required *in vivo* for *A. phagocytophilum* adaptation to the tick. Mice were infected with either the intergenic control or the *tr1*::Himar1 mutant *A. phagocytophilum*. Seven days post-infection, bacterial burden in the mice was quantified and found to be equivalent between the strains by relative gDNA copy number, indicating that *tr1* is not required during murine infection (Fig. 2a). Burden-matched mice from these groups were used to feed *I. scapularis* larval ticks to repletion. We found ticks that fed on mice infected with *tr1*::Himar1 contained significantly less live *A. phagocytophilum,* by qRT-PCR, than those that fed on mice infected with the control strain (Fig. 2b). These findings indicate that *tr1* is dispensable for murine infection but required for colonization of the arthropod vector.

### Tr1 is a multimeric helix-turn-helix DNA-binding protein

**Fig 3:**
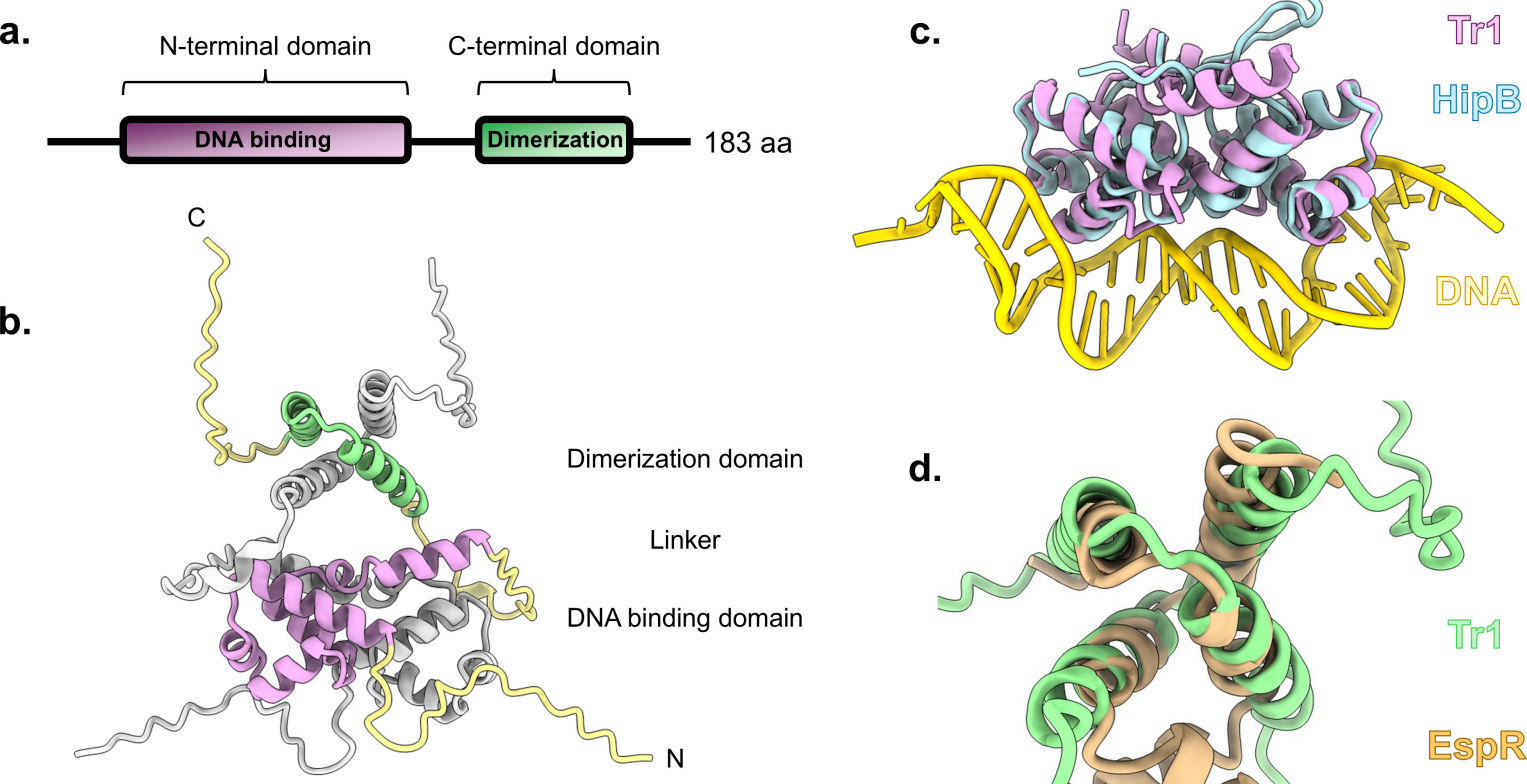
Structural modeling of the Tr1 protein. (**a**) Schematic of the predicted Tr1 protein domain architecture. Ordered domains are displayed as boxes and predicted disordered regions as solid black lines. (**b**) Cartoon representation of a predictive Tr1 dimer protein model. For one Tr1 chain, the N-terminal DNA-binding domain is colored plum, the C-terminal dimerization domain is colored light green, and disordered flanking and linker regions are colored pale yellow. The second Tr1 chain is colored gray. (**c**) Dimeric H-T-H domain of Tr1 (colored magenta) superimposed onto the crystal structure of HipB bound to its DNA operator from *Shewanella oneidensis* (46) (colored light blue; PDB: 4PU4). (**d**) Dimeric C-terminal domain of Tr1 (colored green) superimposed onto the crystal structure of EspR from *M. tuberculosis* (47) (colored brown; PDB: 4NDW).

Having established that Tr1 is important for adaptation to the tick host by *A. phagocytophilum*, we next used bioinformatics approaches to predict the functions of the Tr1 protein. First, a structural model of monomeric Tr1 protein (amino acids 1–183) was generated using ColabFold (45). The resulting predicted Tr1 monomer model is composed of two ordered and predominantly α-helical domains separated by a central disordered peptide linker and flanked by disordered peptides at both the N and C termini (Fig. 3a). In total, the model contains approximately 58 residues of predicted disorder. While the global confidence score for the predicted model was low (pTM = 0.468), the local confidence scores for the two ordered domains were relatively high (pLDDT greater than 80 for residues ~25–90 and ~130–155) (Fig. S1).

Next, we used the Dali server (48) to search the Protein Data Bank (PDB) for structures similar to Tr1 that have been the subject of structure-function analyses. Among the results of the Dali search, nine of the 10 structures most similar to Tr1 were DNA-binding proteins with similar N-terminal domains and function as dimers. Comparing the N-terminal domain of Tr1 with these structures reveals that Tr1 is likely to be a five-helix bundle containing a DNA-binding helix-turn-helix motif (H-T-H), similar to cI/Cro-like transcription factors (Fig. 3a).

Prompted by this observation, we next used ColabFold Multimer to generate a model of dimeric Tr1 protein. In the resulting model, the N-terminal domains of Tr1 are closely associated with one another, forming a symmetrical dimer (Fig. 3b). The C-terminal domains of the two Tr1 monomers, each resembling two antiparallel helices separated by a turn, interdigitate with one another, forming an additional point for dimerization between the two protein chains (Fig. 3b). While the global confidence score for the dimeric model of Tr1 was higher than that of the monomeric model (dimer pTM 0.557), the confidence score for the interface of the dimeric Tr1 model was low (ipTM = 0.545). We considered that the large proportion of disordered protein in Tr1 could skew the interface confidence scores in our predictions, so we tried to predict dimeric models for Tr1 residues corresponding to the N- and C-terminal domains only. Interface confidence scores for the dimeric Tr1 N- and C-terminal domains in isolation were high enough to suggest plausible interactions (piTM = 0.763 and 0.69, respectively).

Structures of dimeric H-T-H proteins identified by our Dali search were superimposed onto the Tr1 dimer model to further assess this predicted structure. The N-terminal domain of Tr1 dimerizes in a manner typical of other dimeric H-T-H proteins, including those bound to DNA (Fig. 3c). Frequently, H-T-H proteins that bind DNA as symmetrical homodimers bind to palindromic DNA sequences, with each protein binding to a half-site of the palindrome. From these structural comparisons, we predict that this is likely to also be the case for Tr1. Fewer structural homologs of the C-terminal domain of Tr1 were identified, but we noted similarity between the Tr1 dimer model and the EspR transcription factor of *Mycobacterium tuberculosis* (Fig. 3d). EspR dimerizes via a domain similar to the Tr1 C-terminal domain, and this is required for EspR to bind DNA with high affinity (49).

**Fig 4:**
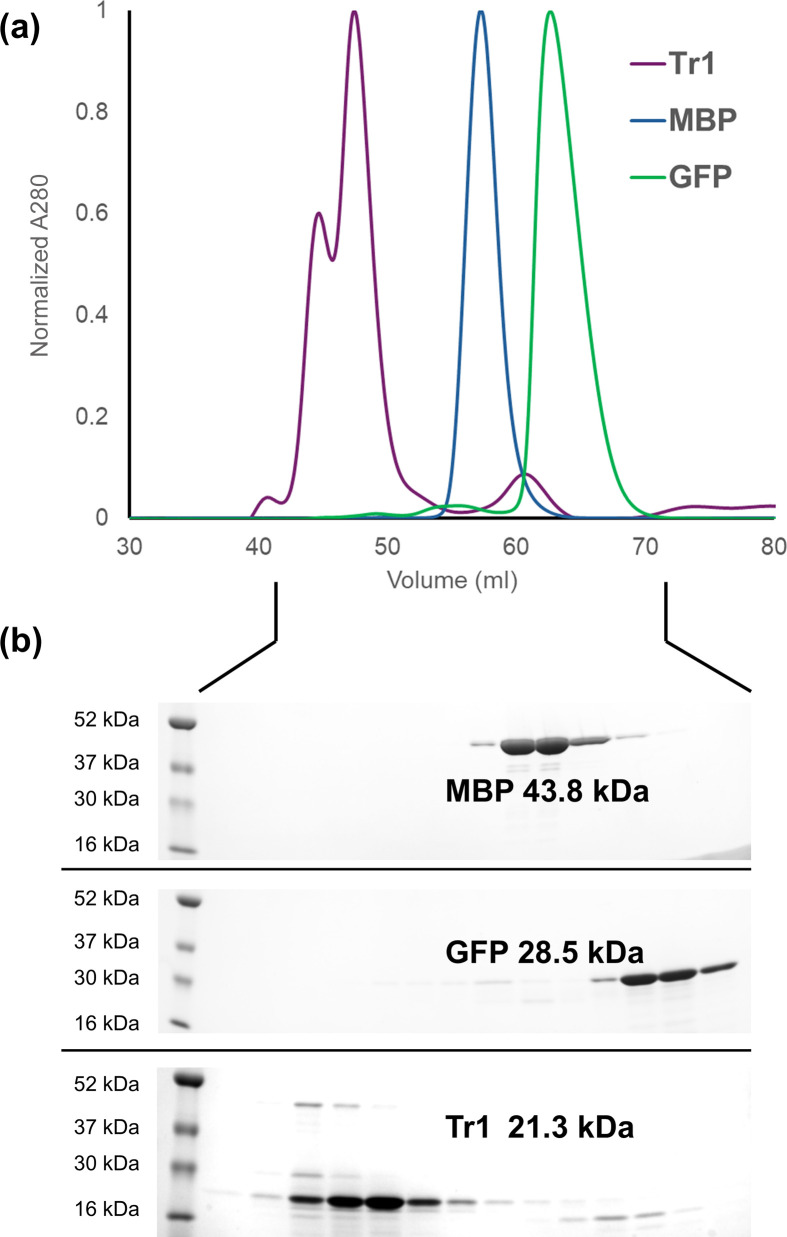
Tr1 forms multimers in solution. (**a**) Chromatograms of Tr1, MBP, and GFP separated by gel filtration. The Tr1 protein lacks tryptophan residues and absorbs weakly at 280 nm, so the peaks are shown as normalized absorbance. (**b**) SDS-PAGE gel of fractions collected from equivalent volumes in gel filtration experiments. The left-hand lane of each gel contains molecular weight markers with the known weights labeled.

Next, we used gel filtration chromatography to assess whether purified recombinant Tr1 protein could form dimers or other multimers in solution. Recombinant Tr1 protein was purified, and affinity tags were removed prior to running on a gel filtration column. We reasoned that removing any additional protein sequences from Tr1 would yield protein most similar to that found in its native environment. Since the expected molecular weight of monomeric recombinant Tr1 is 21.3 kDa, we also ran purified MBP and GFP, predominantly monomeric proteins with molecular weights of 43.8 kDa and 28.5 kDa, respectively, as controls. Both MBP and GFP eluted from the gel filtration column as monodisperse peaks, whereas Tr1 eluted as two overlapping peaks, indicating that Tr1 exists in multiple forms in solution (Fig. 4a). SDS-PAGE analysis of fractions collected from these experiments confirmed that the peaks contained the expected proteins (Fig. 4b). Apparent molecular weights for the peaks were calculated with a standard curve based on their elution volumes. The calculated apparent molecular weights for GFP and MBP were 31.6 and 45.7 kDa, respectively, roughly consistent with their expected weights as monomers. The second peak of Tr1 eluted at an apparent molecular weight of 79.4 kDa, suggesting that Tr1 can form tetramers in solution. The first peak of Tr1 eluted at a volume outside of our standard curve and potentially contained aggregated proteins. We generated a predicted tetrameric model of full-length Tr1, but global confidence scores were noticeably lower than those for other Tr1 models (pTM 0.414), so we did not present these data here. Further biochemical data are required to guide additional predictive modeling of Tr1 multimers.

Taken together, we predict that Tr1 is composed of H-T-H and dimerization/multimerization domains joined and flanked by regions of disorder. Comparisons indicate that Tr1 might have similarity to cI/Cro transcription factors and EspR from *M. tuberculosis*. Gel filtration experiments demonstrate that Tr1 forms multimers in solution, supporting these predictions; however, a high-confidence predicted model of a Tr1 tetramer could not be generated.

### Tr1 binds the promoters of neighboring genes omp1X and omp1N

**Fig 5:**
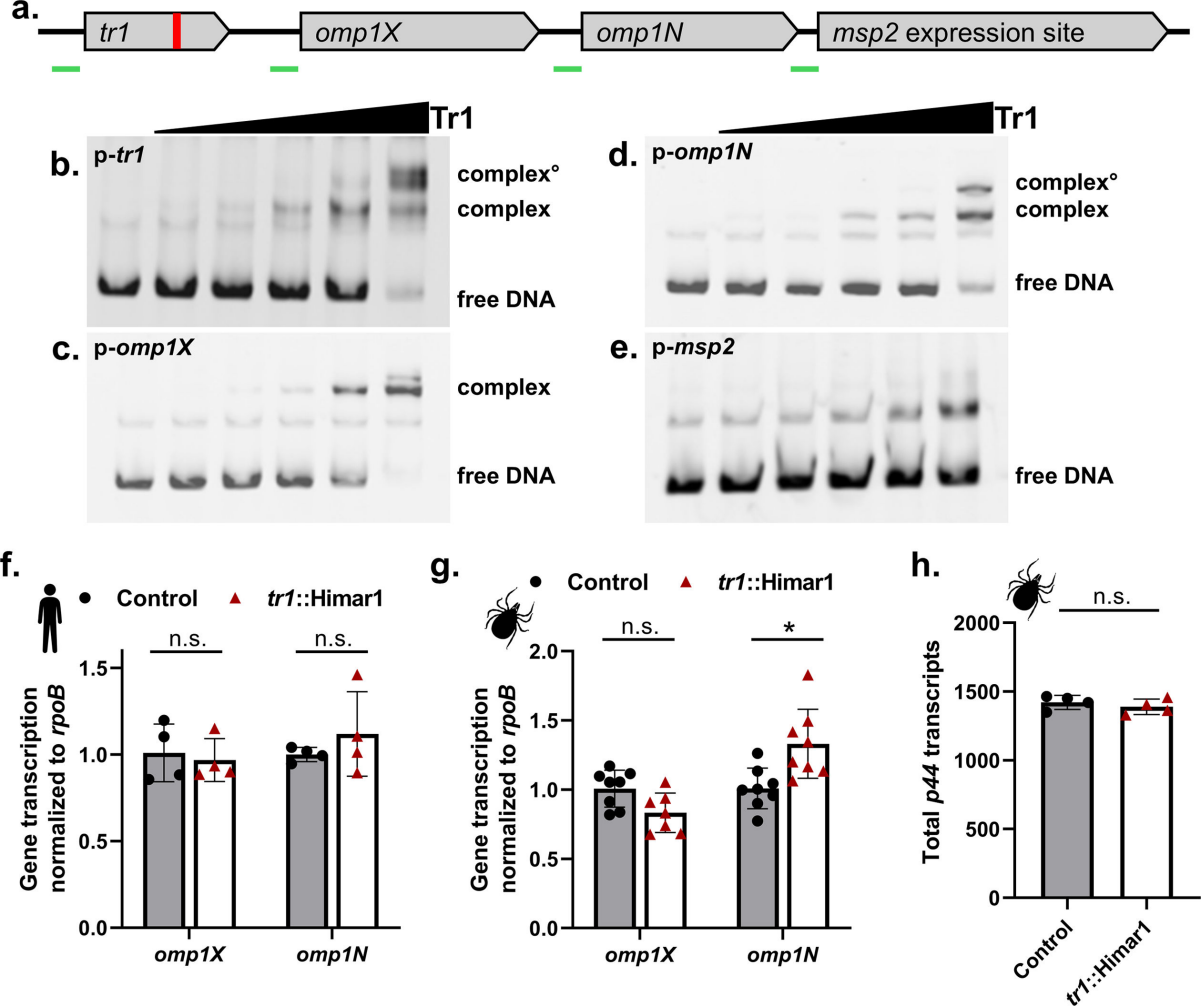
Tr1 binds promoters of neighboring *omp1X* and *omp1N*. (**a**) Diagram of *tr1* gene with downstream neighboring outer membrane protein genes *omp1X*, *omp1N*, and the *msp2/p44* expression site. (b–e) EMSA shifts with increasing rTr1 (0, 0.0625, 0.125, 0.25, 0.5, and 1 μM) with DNA probes of (**b**) *tr1*, (**c**) *omp1X*, (**d**) *omp1N*, and (**e**) *msp2/p44* promoter sequences. (**f and g**) Transcription of *omp1X* and *omp1R* from control or *tr1*::Himar1 *A. phagocytophilum* mutant strains at 24 h post-infection in (**f**) human HL60 or (**g**) tick ISE6 cells. Transcripts measured by qRT-PCR and normalized to *rpoB* via ΔΔCt. Data displayed as mean with ± SD of four replicate infections measured with two technical replicates each. *P < 0.05 (Mann-Whitney *t*-test). (**h**) *msp2/p44* transcripts totals across all gene variants sequenced by RNA-seq from *tr1*::Himar1 or control *A. phagocytophilum* infected ISE6 tick cells. Bars are mean ± SD of four independent infections, each shown as points. n.s. > 0.05 (Mann-Whitney *t*-test).

The *tr1* gene was first identified in studies examining the expression of downstream genes *omp1X* and *omp1N* and the *msp2/p44* expression site (43, 44) (Fig. 5a). We tested Tr1-binding upstream of *tr1*, *omp1X*, *omp1N*, and the *msp2/p44* expression locus by electrophoretic mobility shift assays (EMSAs) using DNA probes for sequences preceding each gene (Fig. 5a). EMSA shifts indicated that Tr1 complexed with promoters p-*tr1* (Fig. 5b), p-*omp1X* (Fig. 5c), and p-*omp1N* (Fig. 5d). At higher Tr1 concentrations, p-*tr1* and p-*omp1N* also displayed secondary complexes, suggesting multiple Tr1-binding sites or higher-order Tr1 oligomer complexes (Fig. 5b and d). DNA sequence upstream of the *msp2* expression locus did not shift at any concentration of the Tr1 protein, indicating that Tr1 does not individually regulate *msp2* (Fig. 5e).

To determine how loss of *tr1* affected transcription of these genes, RNA was collected from control and *tr1*::Himar1 *A. phagocytophilum*-infected ISE6 cells. Surprisingly, qPCR measuring *omp1X* and *omp1N* transcription from *tr1*::Himar1 and control *A. phagocytophilum* strains (Fig. 5f and g) found that only *omp1N* displayed a small but significant expression difference during tick cell infection (Fig. 5g), suggesting that Tr1 binding alone does not fully account for the behavior of these genes.

### tr1 is required for *A. phagocytophilum* transcriptional shift during tick cell infection

Tr1’s predicted role as a transcriptional regulator (43, 50) and the inability of the *tr1*::Himar1 to survive in tick cells led us to investigate how the loss of *tr1* affects the transcriptome during tick cell infection using RNA sequencing (RNA-seq). ISE6 tick cells were infected with *A. phagocytophilum tr1::Himar1* or the control strain. Our previous qPCR for *A. phagocytophilum* gDNA from infected ISE6 cells found that the *tr1*::Himar1 strain persists up to day 3 before declining relative to the control strain (Fig. 1c). Since measuring genomic DNA (gDNA) may also reflect dead bacteria, we measured *A. phagocytophilum 16S* RNA relative to *I. scapularis actin* transcripts as an indicator of bacterial survival at 12, 24, and 48 h post-infection (hpi). At 24 h, *tr1*::Himar1 survival was 63% of the control strain but fell to only 10% by 48 hpi (Fig. S2). To capture transcriptional differences when *tr1*::Himar1 bacteria remained viable, we performed RNA-seq at 24 hpi.

Genes downstream of *tr1*, *omp1X*, *omp1N*, and *msp2*^43^ (Fig. 5a) were all measured in the RNA-seq data. Similar to the qRT-PCR measurements (Fig. 5g), *omp1X* did not significantly differ between *tr1*::Himar1 and the control strain. *omp1N* had a small but significant increase in expression relative to the control (Table S1). Measuring *msp2* transcription is complicated by the presence of multiple *msp2* pseudogenes, which *A. phagocytophilum* uses to evade the mammalian antibody response through antigenic variation. For individual *msp2* variants to be transcribed, the pseudogenes recombine into the expression site adjacent to *omp1N* (43, 44, 51–54) (Fig. 5a). In our RNA-seq data, 97 *msp2* variants were detected, with 15 showing significant transcription differences between *tr1*::Himar1 and the control strain (Table S2). To quantify total *msp2* transcription from the expression site, we summed all *msp2* transcripts from the RNA-seq data. This found no difference in overall *msp2* transcription between the *tr1*::Himar1 and control strain (Fig. 5h). Expression differences among individual *msp2* variants likely reflect an *msp2* diversity bottleneck during Himar1 library construction and/or *msp2* recombination, since the *tr1*::Himar1 and control strains were purified from the larger collection. These findings again suggest that the importance of *tr1* extends beyond its immediate genetic neighborhood.

Beyond *tr1*’s immediate neighbors, RNA-seq identified 177 genes as differentially expressed between *tr1*::Himar1 and the control *A. phagocytophilum* strain (adj. *P* value < 0.05) during tick cell infection (Table S1). Aside from *msp2* variants, 22 genes were >2-fold upregulated in the *tr1*::Himar1, although none have known host or vector-specific expression (32, 33) and are largely ribosomal or other core bacterial genes (34). Conversely, 14 genes had >2-fold reduced transcription from the *tr1*::Himar1 mutant (Table 1). Seven of these (HGE1_03907/APH_0916, *ateA*, *tr1*, HGE1_01872/APH_0406, *msp4*, HGE1_04767/APH_1111, HGE1_03162/APH_0720) have known tick-specific expression patterns (10, 32, 33), which indicates that *tr1* is needed to upregulate *A. phagocytophilum* genes specific for the arthropod vector.

**TABLE 1:** Genes measured by RNA-seq with ≥2-fold reduced expression in *tr1*::Himar1 mutant during tick cell infection

Gene or locus_tag	Note	Base mean	Fold change	Adj. *P* value	Tn mutant
HGE1_03907	Putative T4SS effector	11	8.2	0.00	
HGE1_02492	T4SS effector AteA	25	7.9	0.00	Y^*a*^
*tr1*	HTH DNA binding	121	5.7	0.00	Y
HGE1_04267	Unknown	93	4.7	0.00	
HGE1_01872	Membrane porin	15	3.9	0.00	Y
*msp4*	Major surface protein	31	3.9	0.00	
HGE1_00340	rRNA methyltransferase	9	3.5	0.01	
HGE1_00460	Unknown	15	2.7	0.01	
HGE1_04767	*mrp*/NBP35 family	12	2.6	0.02	
HGE1_03162	Unknown	49	2.6	0.00	Y
HGE1_04257	*dnaA*/*hda* regulator	16	2.3	0.03	
HGE1_01837	ABC transporter	19	2.1	0.01	
*nrdR*	Transcription regulator	14	2.1	0.04	
HGE1_06057	Unknown	431	2.0	0.00	Y

^*a*^ Y indicates Himar1 transposon mutants disrupting the indicated gene were identified in the previously published mutant library (34).

### Tr1 binds promoters of genes of tick-specific *A. phagocytophilum* genes

**Fig 6:**
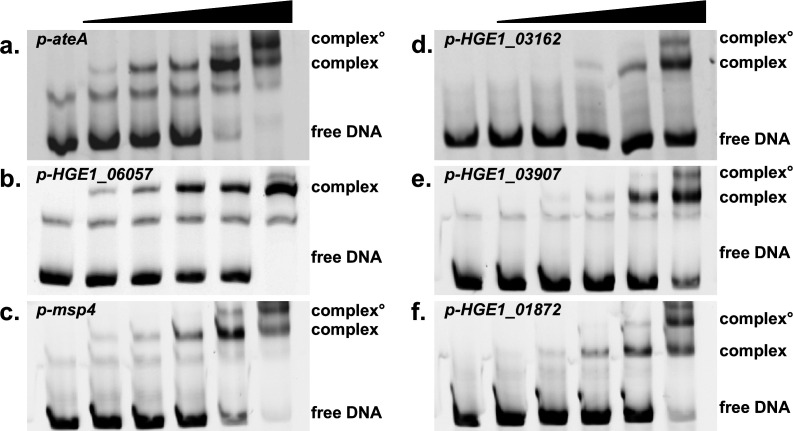
Tr1 binds the promoters of tick-specific *A. phagocytophilum* genes. EMSA shifts with increasing rTr1 (0, 0.0625, 0.125, 0.25, 0.5, and 1μM) tested against promoter sequences of (**a**) *ateA*, (**b**) *HGE1_06057*, (**c**) *HGE1_01872*, (**d**) *HGE1_03162*, (**e**) *HGE1_03907*, and (**f**) *msp4*.

We next asked if Tr1 binds promoters of any tick-specific genes impacted by the *tr1*::Himar1 mutation. EMSAs were performed with increasing Tr1 protein against promoter sequences preceding *ateA*, HGE1_06057/APH_1380, HGE1_03162/APH_0720, HGE1_03907/APH_0916, HGE1_01872/APH_0406, and *msp4* (Fig. 6). Tr1 shifted probes for *ateA* (Fig. 6a), HGE1_06057 (Fig. 6b), and *msp4* (Fig. 6c) at all concentrations, with the majority of the probe band shifted at the highest. HGE1_03162 (Fig. 6d), HGE1_03907/APH_0916 (Fig. 6e), and HGE1_01872/APH_0406 (Fig. 6f) probes also shifted at Tr1 concentrations ≥ 0.125 µM. Similar to EMSAs with probes for *tr1*, *omp1X*, and *omp1N*, the higher Tr1 concentrations produced secondary shift bands, suggesting higher-order complexes. These findings demonstrate direct Tr1 interaction with promoters for tick-specific *A. phagocytophilum* genes. These EMSA results and the differential expression of these genes by the *tr1*::Himar1 mutant strain indicate direct regulation by Tr1.

### Tr1 regulated genes are important for *A. phagocytophilum* survival in tick cells

**Fig 7:**
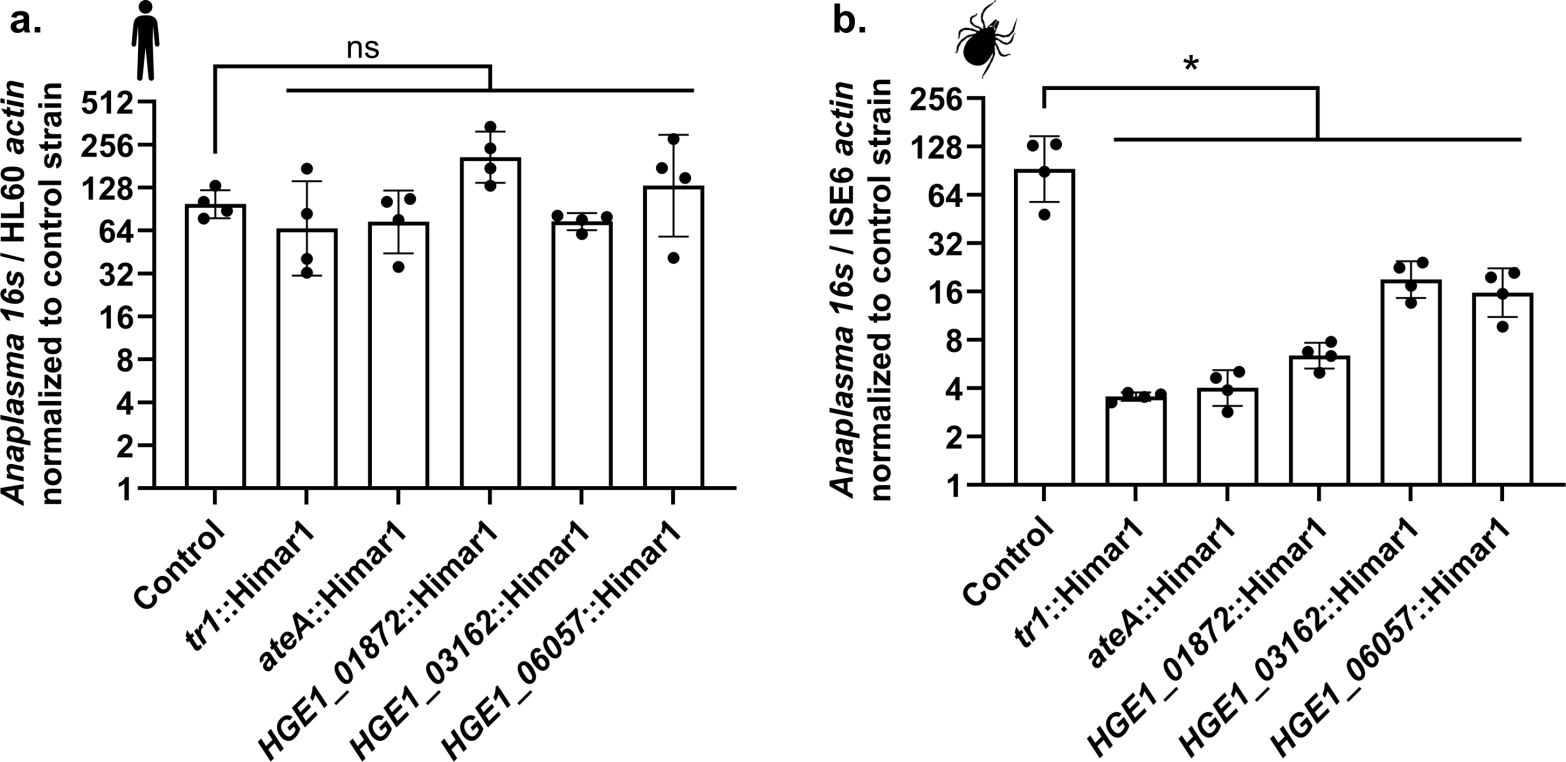
Tr1 impacted genes are necessary for survival during tick cell infection. Survival of indicated Himar1 transposon mutant *A. phagocytophilum* strain relative to intergenic control::Himar1 strain in (**a**) human HL60 (48 hpi) and (**b**) tick ISE6 cells (eight dpi). Bacterial burden measured by qRT-PCR of *A. phagocytophilum 16S* vs *Ixodes* actin transcripts. Data displayed as mean ± SD of four replicate infections measured in two technical replicates each. Graph representative of three replicate experiments. * <0.005 (Mann-Whitney *t*-test).

Inability of the *tr1*::Himar1 to survive in tick cells and the reduced expression of known tick-specific *A. phagocytophilum* genes led us to ask whether these genes are important for tick cell infection. We previously showed that *ateA*::Himar1 was similarly defective for survival in tick cells and whole ticks (10). From the *A. phagocytophilum* Himar1 mutant library, we identified and isolated three additional mutants disrupted in genes putatively regulated by Tr1: *HGE1_01872*, *HGE1_03162*, and *HGE1_06057*. Growth of all mutant strains in human HL60 cell culture was comparable to the control strain (Fig. 7a). Accordingly, we infected ISE6 tick cells with *A. phagocytophilum* strains *tr1*::Himar1, *ateA*::Himar1, *HGE1_01872*::Himar1, *HGE1_03162*::Himar1, *HGE1_06057*::Himar1, and intergenic control::Himar1. At 8 days post-infection, all tested strains had significantly reduced *Anaplasma* burden in the tick cells relative to the intergenic Himar1 control, with *tr1::Himar1* being the most severe (Fig. 7b). From this, we demonstrated three additional tick-specific genes HGE1_01872, HGE1_03162, and HGE1_06057 as critical for infecting ticks. Our results suggest that the tick-specific defect of the *tr1*::Himar1 mutant strain stems from its failure to express genes controlled by Tr1 that are critical for adaptation to the tick cell environment.

### tr1 is necessary for tick-specific alterations in the T4SS apparatus

The promoter sequences of *ateA* and HGE1_06057 had high affinity for Tr1 by EMSA (Fig. 6a and b). We previously identified AteA as the first *A. phagocytophilum* T4SS-secreted effector specific to tick infection (10). HGE1_06057 is also predicted as a T4SS substrate by multiple effector prediction algorithms (12, 55). Tr1 binding to promoters of effectors prompted us to ask if Tr1 influences expression of T4SS machinery itself.

**Fig 8:**
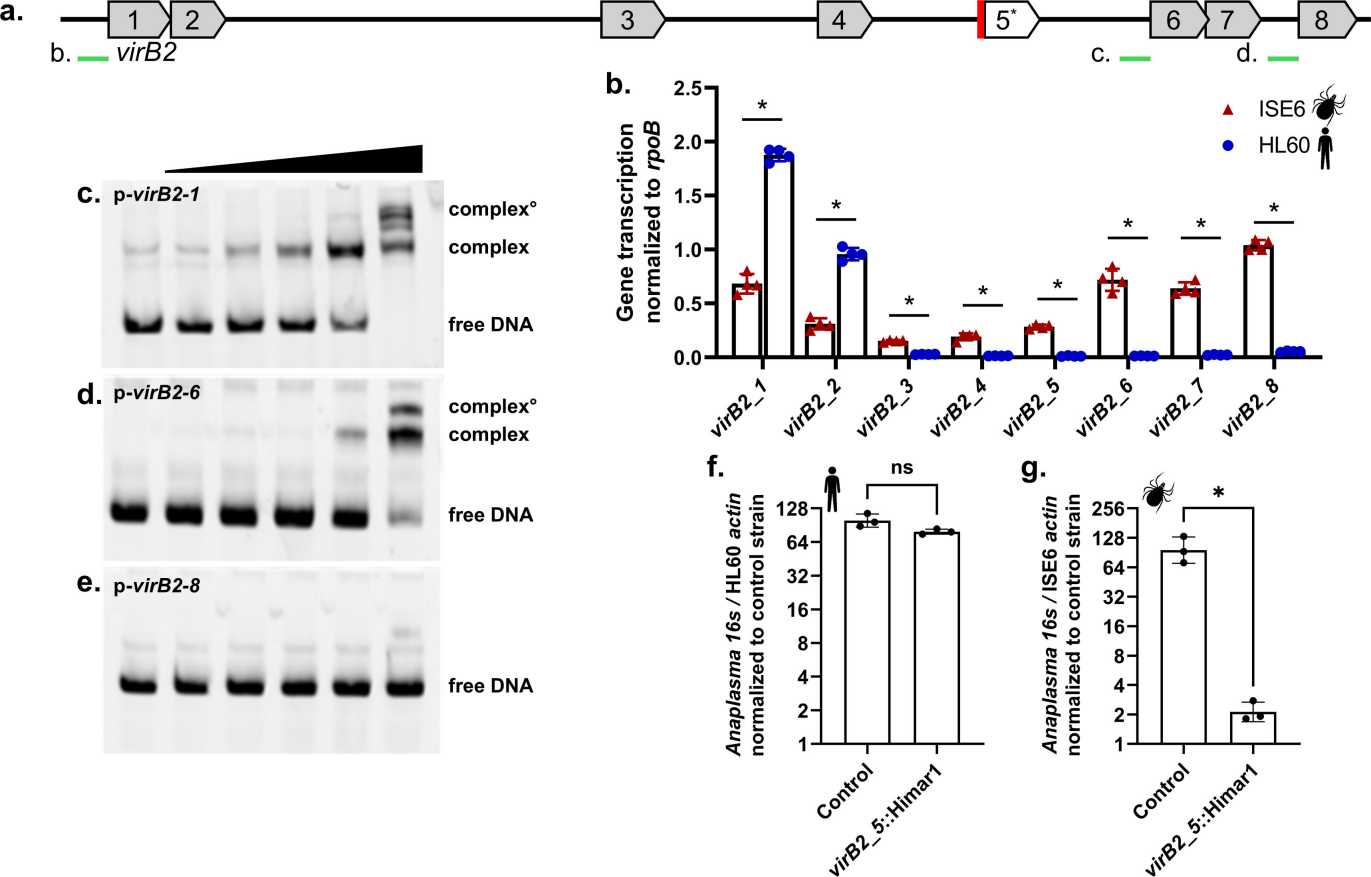
Tr1 is required for tick-specific remodeling of the T4SS pilus. (**a**) Diagram of the *virB2* paralog genes (locus HGE1_04947 – HGE1_04867), the location of the *virB2_5*::Himar1 insertion mutation (red), and potential promoter regions subject to EMSA (green). (**b**) Transcription of the *virB2* genes during wild-type *A. phagocytophilum* infection of mammalian HL60 and tick ISE6 cells measured by qRT-PCR. Data shown as mean ± SD of four biological replicates. (**c, d, e**) EMSA shifts with increasing rTr1 (0, 0.0625, 0.125, 0.25, 0.5, and 1 μM) with putative promoter sequences upstream of (**c**) *virB2_1*, (**d**) *virB2_6*, and (**e**) *virB2_8*. (**f and g**) Growth of *A. phagocytophilum virB2_5*::Himar1 or control strain in cell culture infections of (**f**) human HL60 cells and (**g**) tick ISE6 cells. *A. phagocytophilum* burden measured by bacterial gDNA relative to eukaryotic host gDNA via qPCR. Data displayed as mean with ± SD of three biological replicates with two technical replicates each. Data are representative of three experimental replicates. *P < 0.05 (Mann-Whitney *t*-test).

An oddity of the *Anaplasma* T4SS is that some components of the apparatus are encoded by multiple paralogs (16, 23). In particular, the needle-like pilus of the *A. phagocytophilum* T4SS is encoded by eight paralogs of the pilin gene *virB2* (Fig. 8a). Transcriptomics studies noticed that different combinations of *virB2* paralogs are upregulated when *A. phagocytophilum* infects mammalian or tick cells (32, 33). Because tiling arrays used in these studies can conflate signal among paralogs with close sequence identity (33), we first validated *virB2* expression. We designed primers specific to each *virB2* paralog. We grew *A. phagocytophilum* in human HL60s or tick ISE6 cells and measured transcription of each *virB2* paralog by qRT-PCR. As in the transcriptomic studies, mammalian cell-cultured *A. phagocytophilum* expressed only the *virB2* paralogs *virB2_1* and *virB2_2* (Fig. 8b). Conversely, transcription of *virB2_1* and *virB2_2* was reduced during growth in tick cells, while *virB2*_3 to virB2_8 were upregulated (Fig. 8b). To examine if the tick-specific expression pattern among the *virB2* paralogs is dependent on *tr1*, we reviewed our RNA-seq findings for the *tr1*::Himar mutant. Expression of *virB2_1* and *virB2_2* expression was significantly elevated (1.47- and 1.38-fold) in the *tr1*::Himar1. While not significant due to low number of total reads, *virB2_6* and *virB2_7* had reduced expression (0.55- and 0.218-fold) in *tr1*::Himar1 relative to the control strain (Table S1). Together, this indicated that the *tr1*::Himar1 strain fails to adjust expression among the *virB2* paralogs during tick cell infection and retains a *virB2* expression pattern similar to mammalian infection.

### Differentially expressed T4SS virB2 paralogs are bound by Tr1

We asked if Tr1 can directly bind promoter regions among the differentially expressed *virB2* paralogs. DNA probes designed upstream of *virB2_1*, *virB2_6*, and *virB2_8* (Fig. 8a) were tested with increasing concentrations of Tr1 protein by EMSA. Tr1 shifted the promoters of both *virB2_1/2* (Fig. 8c) and *virB2_6/7* (Fig. 8d), with *virB2_1/2* shifting completely at the highest concentration. As with other targets, possible secondary complexes were also visible, suggesting possible higher-order interactions. Minimal shifting was seen with probes designed upstream of *virB2_8* (Fig. 8e), suggesting either independent regulation or co-regulation in an operon with upstream genes *virB_6/7*.^56^ Our findings indicate that Tr1 directly participates in regulation of tick-specific expression patterns of T4SS components.

### Tick-specific virB2 paralogs are necessary for *A. phagocytophilum* survival in tick cells

As with the other Tr1-regulated targets, we asked whether the tick-specific *virB2* paralogs are necessary for *A. phagocytophilum* survival in tick cells. From the transposon mutant library, we obtained a mutant strain disrupted in *virB2_5* (*virB2_5*::Himar1) (Fig. 8a). In the *A. phagocytophilum* HGE1 strain genome (APHH00000000), *virB2_5* is not annotated and therefore was not initially mapped by RNA-seq. However, we do detect transcription from the *virB2_5* locus during tick cell infection (Fig. 8b). The *virB2_5*::Himar1 mutant grew as well as the control strain in human HL60 cells (Fig. 8f). *A. phagocytophilum virB2_5*::Himar1 and the intergenic control::Himar1 strain were collected from HL60 cell culture and used to infect ISE6 tick cells. Eight days post-infection, the *Anaplasma* burden of *virB2_5*::Himar1 was greatly reduced relative to the control::Himar1 strain in tick cells (Fig. 8e). This finding indicates that tick-specific *virB2* paralogs are important for *A. phagocytophilum* adaptation to the tick.

### Tr1 homologs are found throughout the Rickettsiales

Having established a role for Tr1 in the survival of *A. phagocytophilum* within the tick host, we queried whether Tr1 homologs are present in other Rickettsiales (43), since many of these bacteria also have life cycles that involve cycling between vertebrate and arthropod hosts. Using sequence-based searches within the Rickettsiales, we identified candidate homologs of Tr1 in diverse Anaplasmataceae genomes. The *tr1* genes of *Anaplasma*, *Ehrlichia*, and *Neoehrlichia* share a high degree of genomic synteny and are invariably co-localized with *ndk* (encoding nucleoside-diphosphate kinase) and one or more OMP-encoding genes. In *Wolbachia* and “*Candidatus* Mesenet,” the *tr1* locus is more variable, with *ndk* being lost and OMP-encoding genes co-localized at the *tr1* locus in some, but not all, species.

We used Foldseek to extend our search for more distant Tr1 homologs, filtering our results for Rickettsiales bacteria. Using this approach, we identified Tr1 proteins in *Rickettsia* and “*Candidatus* Tisiphia” species. While the *tr1* locus in *Rickettsia* species is not syntenic with those from the Anaplasmataceae, the *Rickettsia tr1* gene is most often co-localized with *sca4*. Intriguingly, *sca4* has been identified as having a role in fitness of *Rickettsia parkeri* within the tick host (57). Yet more distant Tr1 homologs were also identified in *Midichloria* and *Orientia*, although in the latter, the C-terminal domain is truncated or lost. Candidate *tr1* homologs are co-localized with genes that encode proteins with unknown functions in many Rickettsiales.

These results indicate that Tr1 represents a family of transcription factors found broadly across the Rickettsiales, suggesting that some of our findings could be extrapolated to other bacteria of this order.

## DISCUSSION

As a vector-borne pathogen, survival in both the tick and the mammal is essential to complete its transmission cycle of *A. phagocytophilum* and be maintained in the environment. We know the bacteria undergo extensive transcriptional changes between the two environments (32, 33) and have identified individual adaptations that are critical for either mammalian or tick infection (34–37). Understanding how rickettsial pathogens switch their host tropism to the arthropod could inform interventions to disrupt the transmission cycle. In this work, we identified Tr1 as a critical switch regulating *A. phagocytophilum* adaptation to the tick and identified Tr1-controlled genes necessary for survival in the arthropod. Collectively, our findings uncover a central node in a network of changes necessary for rickettsial bacteria to complete their vector-borne lifecycle.

Due to the obligate intracellular lifecycle and lack of compatible extrachromosomal plasmids, genetic manipulation of *A. phagocytophilum* remains challenging. The Himar1 transposon library has proven to be an invaluable resource, allowing the first phenotypic testing of gene disruptions in *Anaplasma* (34). The library was generated in HL60 cell culture, which prohibits mutation in genes essential for survival in mammalian cells. However, *A. phagocytophilum* genes specific to tick infection were readily mutated (34, 35). Such mutants have been leveraged to uncover tick-specific roles of the T4SS effector AteA (10), the duplicate T4SS component VirB6-4 (36), and the surface protein-modifying O-methyltransferase (37). In our study, we extensively relied on mutants from this library to uncover tick-specific contributions of *tr1* and five of the genes it impacts. Until more targeted genetic tools are available in this system, the *A. phagocytophilum* Himar1 transposon library remains a critical resource.

Initial interest in the *tr1* gene stemmed from its location upstream of outer membrane protein genes *omp1X* and *omp1N* and the *msp2/p44* expression site (43, 44). ApxR was identified as the first *Anaplasma* DNA-binding transcriptional regulator and was later shown to bind its own promoter and upstream region of the *p44/msp2* expression site. Similarly, the ApxR homolog EcxR in the related rickettsial pathogen *Ehrlichia chaffeensis* binds promoters of *tr1* and downstream outer membrane protein genes (58, 59). *E. chaffeensis* Tr1 also binds upstream membrane protein genes *p28* and *omp-1B* (58). We found that *A. phagocytophilum* Tr1 binds DNA sequences upstream of *omp1X* and *omp1N*. However, only expression of *omp1N* differed between the *tr1*::Himar1 mutant and control strain, indicating that *tr1* is not the only factor controlling these genes. The relatively small change in *omp1N* may also reflect indirect effects of the mutant’s reduced fitness in tick cells. Although the number, identity, and arrangement of adjacent membrane protein genes downstream of *tr1* in *Anaplasma* and *Ehrlichia* differ greatly (44, 58), common models may emerge showing how Tr1 and ApxR/EcxR participate in the regulation of the *tr1* adjacent surface protein genes.

Looking past its immediate chromosomal neighbors, multiple tick-specific membrane protein genes had reduced expression in the Himar1::*tr1* and were directly bound by Tr1. These include *HGE1_03162* (*APH_0720*), *HGE1_01872* (*APH_0406*), *msp4*, and *HGE1_03907* (*APH_0916*). Transcription of all four is highly specific to *A. phagocytophilum* infection in tick cells (32, 33). Reflecting the expression pattern, we found that *HGE1_03162* and *HGE1_01872* are necessary for tick cell infection. Adjacent to *HGE1_01872*, *A. phagocytophilum* encodes two *HGE1_01872* paralogs *asp62* (*HGE1_01862*, *APH_0404*) and *asp55* (*HGE1_01867*, *APH_0505*). Antibodies against Asp62 and Asp55 were found to limit *A. phagocytophilum* infection of human HL60 cells (60). While Asp62 and Asp55 are expressed during both mammalian and tick cell infection, HGE1_01872 is specific to the arthropod vector (32, 33). Both HGE1_01872 and Msp4 are substrates of an O-methyltransferase required for tick infection and promote binding to *Ixodes* cells (37, 61, 62). Similar to HGE1_01872, HGE1_03907 (APH_0916) is adjacent to the invasion protein gene *aipA*, which is necessary for infection of mammalian cells (63); however, sequence identity does not suggest homology. These findings implicate Tr1 as the switch regulator governing this remodeling of the *A. phagocytophilum*’s surface proteome necessary for tick infection.

Beyond surface protein genes, Tr1 plays a broader role in controlling genes expressed during infection of the tick. Our findings identify Tr1 as a critical regulator of tick-specific effector AteA and specialization of the T4SS VirB2 pilus necessary for adaptation to the tick. The full repertoire of effectors secreted by *A. phagocytophilum* is unknown, but effector-predicting algorithms propose as many as 49 (12, 55). So far, few have been experimentally examined (17, 21, 22, 24, 64–66), with only AteA investigated in the context of the arthropod vector (10). The *E. chaffeensis* and *Ehrlichia ruminantium* ApxR homologs, EcxR, were also shown to regulate components of the T4SS apparatus during mammalian cell infection (67, 68). However, EcxR has yet to be examined during tick infection, and binding to effector to *virB2* pilin genes is untested. Uncovering the full regulome of Tr1 and how it works with other regulators to manage adaptation between the mammalian and tick environments will likely require larger unbiased binding and regulation studies.

How does Tr1 recognize its DNA targets and regulate gene expression? Bioinformatic analysis indicates that the Tr1 protein has similarity to H-T-H transcription factors and, inferred from this and our protein modeling, is likely to dimerize and bind palindromic DNA motifs to exert influence on transcription on downstream genes. Consistent with this, we also identified a putative dimerization domain in the C-terminus of Tr1, and we observed that recombinant Tr1 protein forms multimers in solution, probably tetramers and possibly other higher-order structures. Other H-T-H proteins such as the λ repressor share this ability to form multimers of dimers, enabling them to bind to multiple DNA sites cooperatively (69, 70). Indeed, multiple shifts of Tr1-DNA complexes in our EMSAs support the possibility that binding of Tr1 may be cooperative at some of its target sites. Understanding whether or how the multimerization of Tr1 is regulated during infection, the interplay between Tr1 and other transcription factors at promoter regions, and the identification of a consensus DNA target site and regulon will be crucial for determining how Tr1 mediates *A. phagocytophilum* adaptation to the arthropod host.

We identified candidate Tr1 homologs throughout the Rickettsiales. In both *Anaplasma* and some *Rickettsia*, the Tr1 gene is co-localized with genes that are either differentially expressed between the tick and vertebrate host or have been implicated in survival in tick cells. This functional conservation of the *tr1* loci between these two genera makes it interesting to consider that Tr1 might have a role in transcriptional remodeling that underpins cycling between vertebrate and arthropod hosts in diverse Rickettsiales. However, this leaves the question of what the role of Tr1 is in Rickettsiales that survive exclusively within arthropods, such as *Wolbachia*. Furthermore, in many Rickettsiales, the *tr1* gene is co-localized with genes of no known function. While genomic synteny suggests that Tr1 fulfills similar roles in *Anaplasma*, *Ehrlichia*, and *Neoehrlichia*, further study is required to determine how Tr1 functions across diverse Rickettsiales.

Altogether, our work identifies Tr1 as an essential master regulator that remodels the *A. phagocytophilum* transcriptome to infect the tick vector. Further, we identified multiple Tr1 regulated genes that contribute to tick cell infection. With most studies focusing on how *A. phagocytophilum* infects mammals; this work provides insights into an understudied aspect of pathogen biology, uncovering wide-reaching transcriptional events that underpin adaptation to the arthropod vector. Unable to be transmitted vertically to host progeny, *A. phagocytophilum* must cycle between the host and tick vector to be maintained in the environment. Tr1 and the genes under its control are an essential part of this system. Uncovering how *A. phagocytophilum* and other rickettsial bacteria achieve this is key to developing interventions to interrupt the transmission cycle.

## MATERIALS AND METHODS

### Bacterial and eukaryotic cell culture

*Escherichia coli* used for plasmid construction, plasmid amplification, and protein expression was cultured with solid and liquid lysogeny broth (LB) medium with the addition of kanamycin or zeocin (25 µg/mL) antibiotics as needed to select for plasmid-encoded resistance genes. For production of recombinant Tr1 proteins, *E. coli* strain DE3 (New England Biolabs) was used with LB or Terrific Broth (TB) supplemented with 100 µg/mL kanamycin.

HL60 human promyelocytic cells (ATCC; CCL-240) were maintained in Roswell Park Memorial Institute (RPMI) 1640 medium with 10% FBS and 1× Glutamax. HL60 cultures were kept at 37°C with 5% CO_2_ in a humidified incubator. HL60 cell cultures were limited to <20 passages to prevent phenotypic drift and kept between 5  ×  10^4^ and 1  ×  10^6^ cell/mL to prevent differentiation.

Wild-type *A. phagocytophilum* strain HGE1 and HGE1-derived Himar1 transposon mutants were grown in HL60 cells as previously described (34). All Himar1 mutant strains were obtained from the previously established *A. phagocytophilum* mutant collection (34). Status of *A. phagocytophilum* infections in HL60 cells was monitored by Diff-Quick Romanowsky–Giemsa staining. To generate host cell-free *A. phagocytophilum*, peak-infected HL60 cultures (>95% infected cells) were gently sonicated in 5 mL of medium three times for 30 s at 30% amplitude in a water cup sonicator to disrupt HL60 cell membranes. Liberated bacteria were separated from host cell debris by centrifugation at 710 × *g* for 5 min at 4°C. *Anaplasma* numbers were estimated as previously described (71, 72).

*I. scapularis* (Say) embryonic tick cells (ISE6) were maintained in L15C-300 medium with 10% FBS (Sigma; F0926), 10% tryptone phosphate broth (TPB; BD; B260300), and 0.1% lipoprotein cholesterol concentrate (MP Biomedicals; 219147680) (73), in sealed, tissue culture-treated flasks, and incubated at 34°C (74). Infected ISE6 cell cultures were additionally supplemented with 0.25% NaHCO_3_ and 25 mM HEPES buffer (Sigma), cultured in vented flasks, and kept at 34°C in a humidified chamber with 4% CO_2_.

### *A. phagocytophilum* mutant growth curves

*A. phagocytophilum* burden in HL60 and ISE6 cells was evaluated as previously described (36, 37). Briefly, HL60 cells were seeded to 24-well plates at 5 × 10^4^ cells/well and infected at an MOI of 1. Three to four replicate wells were harvested at the time of inoculation and tested time post-inoculation. ISE6 cells were seeded to 24-well plates at 3 × 10^5^ cells/well. After ISE6 cells adhered to the plate for at least 6 h, they were inoculated at an MOI of 50 with host cell-free *A. phagocytophilum* purified from HL60 cultures. The high MOI of 50 was used to account for the lower rate of survival and infection when bacteria are transferred between mammalian and tick cell culture systems. Bacteria were allowed to infect the tick cells for 4 h, after which the medium was exchanged three times to remove excess extracellular bacteria. Four replicate wells were collected following media exchange and subsequent time points. Pelleted samples were frozen and later processed for RNA or gDNA using Zymogen Quick-RNA MicroPrep or QIAGEN DNeasy Blood and Tissue kits. Ratios of bacterial and host cell gDNA copies were measured by qPCR as previously described (10, 36, 37). Levels of live *A. phagocytophilum* Himar1 mutant bacteria in each infected ISE6 or HL60 cell sample were measured by qRT-PCR targeting *A. phagocytophilum* 16S rRNA and *I. scapularis* or human *β-actin* transcripts (Table S3) and compared relative to the intergenic Himar1 control strain by ΔΔCt (10, 71, 75).

### Measuring *A. phagocytophilum* gene transcription

HL60 and ISE6 cells in 24-well plates were infected with wild-type *A. phagocytophilum* HGE1 as described above. At 24 h post-infections, four wells were collected and processed for RNA using the Zymo Quick-RNA MicroPrep Kit (ZymoResearch) according to the manufacturer’s protocol for tissue culture samples. cDNA was generated with the Verso cDNA Synthesis Kit (ThermoFisher). Transcripts of *A. phagocytophilum* genes of interest were measured by qPCR using gene-specific primers (Table S3) and SYBR green iTaq universal Supermix (Bio-Rad; 1725125) according to Bio-Rad-specified cycle conditions. Genes of interest were measured relative to the housekeeping gene *rpoB* and compared between human and tick cell infections by ΔΔCt.

### Animal infection

All mice were purchased at six weeks of age from the Jackson Laboratory. Gender-balanced groups of 10 C57BL/6 mice were infected as previously described (10) with either the *tr1*::Himar1 or the intergenic Himar1 mutant *A. phagocytophilum*. The intergenic Himar1 mutant was previously shown to be phenotypically equivalent to wild type (10, 36, 37). Blood was collected 7 days post-inoculation, and *A. phagocytophilum* burden in the blood was measured by qPCR as in prior studies (16S rRNA/mouse *β-actin* (10, 71, 75) (Table S3). Larval *I. scapularis* ticks purchased from Oklahoma State University (Stillwater, OK, USA) were kept at 23°C with >95% humidity and a 16/8-h light/dark photoperiod. Mice with equivalent *Anaplasma* burden were selected from the intergenic Himar1 and *tr1*::Himar1 groups. Each mouse was infested with 200 naïve larval *I. scapularis* and allowed to feed to repletion. Detached replete ticks were individually processed and assayed for *A. phagocytophilum* burden by qRT-PCR measuring 16S rRNA relative to *I. scapularis β-actin* transcripts (Table S3), with comparisons made by absolute quantification (10, 71, 75). All animal use protocols were approved by the Washington State University Institutional Animal Care and Use Committee (ASAF #6630), and the animal housing facilities at Washington State University in Pullman, WA, maintain AAALAC accreditation.

### RNA sequencing

ISE6 cells were seeded to 12-well plates at 5 × 10^5^ cells/well and allowed to adhere for 6 h. The intergenic transposon control and Himar1::*tr1* mutant *A. phagocytophilum* strains were prepared as host cell-free bacteria from >95% infected HL60 cultures immediately before ISE6 infection. Adhered ISE6 cells were infected at an MOI of 100. Twenty-four hours post-infection, the medium was exchanged three times to remove excess extracellular bacteria, and four replicate wells were collected and RNA isolated with Zymogen Quick-RNA MiniPrep kits. RNA was submitted to the WSU Genomics Core for total RNA-seq analysis. Sequencing libraries were prepared using TruSeq Stranded Total RNA Kit, with Ribo-Zero Plus, and libraries sequenced using a HiSeq 2500, and the results were exported as FASTQ files as previously described (76). RNA-seq data were aligned with the *A. phagocytophilum* HGE1 genome (GCF_000478425.1) (77). Transcript quantification and differential gene expression were analyzed using HTSeq and DESeq2 (78).

### Overexpression and purification of recombinant Tr1 protein

The *tr1* open reading frame, codon-optimized for expression in *E. coli*, was synthesized and cloned by Twist Bioscience into a modified pET28a expression vector. The resultant plasmid encoded the Tr1 protein fused at its N-terminus to a hexa-histidine-tagged maltose-binding protein (MBP), with a tobacco etch virus (TEV) protease recognition site between Tr1 and MBP. The plasmid was transformed into *E. coli* DE3 for overexpression of the recombinant Tr1 protein. Briefly, overnight cultures, grown at 37°C in selective LB medium, were used to inoculate flasks of TB. Overexpression of MBP-Tr1 was induced by the addition of 0.5 mM IPTG once cultures had reached an OD_600_ of 0.5–0.6 and then grown for an additional 18 h at 25°C. Cells were harvested by centrifugation and kept at −20°C for short-term storage.

To purify MBP-Tr1 protein, cell pellets were resuspended in Buffer A (20 mM imidazole, pH 8.0; 20 mM Tris-HCl, pH 8.0; 400 mM NaCl; 2 mM beta-mercaptoethanol) and then lysed by sonication. Lysates were clarified by centrifugation and then loaded onto a HisTrap HP purification column (Cytiva). The column was washed extensively with Buffer A prior to elution of the His-tagged MBP-Tr1 with stepwise (10, 20, 100%) washes with Buffer B (as Buffer A but with 400 mM imidazole). Fractions containing MBP-Tr1 were pooled, concentrated by centrifugal filtration, and then further purified by gel filtration on a Superdex 200 column (Cytiva) equilibrated in SEC Buffer (20 mM Tris-HCl [pH 8.0], 250 mM NaCl, and 2 mM beta-mercaptoethanol). Fractions containing MBP-Tr1, which eluted as a single peak from the gel-filtration column, were supplemented with his-tagged TEV protease and incubated at 4°C for 16 hours to cleave the His-tagged MBP protein from Tr1. The resulting protein preparation was passed over a HisTrap HP column which was then washed step (5, 10, 100%) with Buffer B. Tr1 protein typically passed through the HisTrap HP column or eluted in 5% Buffer B washes and were thus separated from the His-tagged TEV and MBP proteins, which eluted in 100% Buffer B. This purification strategy yielded highly pure Tr1 protein lacking any tags.

### Gel filtration

Purified tagless Tr1 protein, green fluorescent protein (GFP), and maltose-binding protein were analyzed by gel filtration using a HiPrep Sephadex 75 16/600 column (Cytiva), with Buffer B. GFP and MBP were expressed using pET vectors (modified pET41c and pET28a, respectively) in *E. coli* DE3 and purified by nickel affinity chromatography or amylose affinity chromatography using standard methods.

### Electrophoretic mobility shift assays

Promoter-proximal DNA sequences for use in electrophoretic mobility shift assay (EMSAs) were amplified by PCR. Primers for this purpose were designed to yield DNA fragments comprising the ATG start codon of the selected gene plus 250 bp of upstream DNA sequence. In each case, the forward primer was appended with an M13 sequence upstream of the primer sequence (5′-GAG CGG ATA ACA ATT TCA CAC AGG). The resulting DNA fragments were separated by agarose gel electrophoresis, and fragments of the anticipated size were excised and purified using QIAquick Gel Extraction Kit. These purified fragments were then used as templates for a secondary PCR using an M13 sequence primer labeled with FAM at the 5′ end in conjunction with the fragment-specific reverse primer used in the primary PCR. The resulting FAM-labeled PCR products were resolved by agarose gel electrophoresis and purified as described above. Sequences of primers used are included in Table S3.

EMSA assays were performed using a modified protocol described elsewhere (79). Briefly, for each DNA fragment, an EMSA mixture comprising 100 ng FAM-labeled DNA fragment, 200 ng of herring sperm DNA, 5% glycerol, 10 mM Tris-HCl (pH 8.0), and 25 mM KCl was prepared, and 8 µL of this mixture was dispensed into a series of tubes. These were then supplemented with 2 µL of purified Tr1 protein at varying concentrations, or with buffer alone, and then mixed by gentle pipetting. Tr1 protein was used at final concentrations of 0.25, 0.5, 1, 2, and 4 µM for all EMSAs presented here. Resulting mixtures were incubated at 25°C for 30 min before being loaded onto 7.5% polyacrylamide gels buffered with 0.25× TBE and 5% glycerol. Gels were run for 60 min at 100 V, and fluorescent images (green channel) were then collected using a ChemiDoc MP Imaging System (Bio-Rad).

### Bioinformatics

Protein structures were analyzed and visualized in Coot (80) and ChimeraX (81). Modeling and the generation of predicted structures were performed using ColabFold and AlphaFold Server with searches for homologs in the PDB were conducted using the Dali Server (45, 48).

## SUPPLEMENTAL MATERIAL

Supplemental figures msphere.00872-25-s0001.docx Figures S1 and S2.

Table S1 msphere.00872-25-s0002.xlsx RNA-seq of *tr1*::Himar1 mutant and intergenic Himar1 control strain during ISE6 tick cell infection.

Table S2 msphere.00872-25-s0003.xlsx RNA-seq data from Table S1 filtered for p44 paralogs.

Table S3 msphere.00872-25-s0004.xlsx Primers.

ASM does not own the copyrights to Supplemental Material that may be linked to, or accessed through, an article. The authors have granted ASM a non-exclusive, world-wide license to publish the Supplemental Material files. Please contact the corresponding author directly for reuse.
