# Supplementary material for: Adaptation of Anaplasma phagocytophilum to the tick vector is controlled by the transcriptional regulator Tr1
Source: mSphere. 2026 Jun 15;11(7):e00872-25. doi: 10.1128/msphere.00872-25 (PMC13410764; doi:10.1128/msphere.00872-25)

Figure S2. Early survival of *tr1*::Himar1 strain during tick cell infection. *A. phagocytophilum* tr1::Himar1 or control strain in cell culture infections of tick ISE6 cells. *A. phagocytophilum* burden measured by bacterial 16S relative to tick *actin* transcripts via qRT‑PCR. Data displayed as mean with ±SD of three biological replicates. Data are representative of three experimental replicates. *P < 0.05 (Mann‑Whitney t‑test).

**Figure S1.** Figure S1**.** Predicted Local Distance Difference Test (pLDDT) and Multiple Sequence Alignment (MSA) plots from ColabFold of Tr1. (a) pLDDT plot shows that local confidence in the Tr1 model is high in the N‑ and C‑terminal domains, supporting the proposal that Tr1 is comprised of two ordered domains. (b) MSA plot showing that the Tr1 N‑ and C‑terminal domains are evolutionarily conserved.


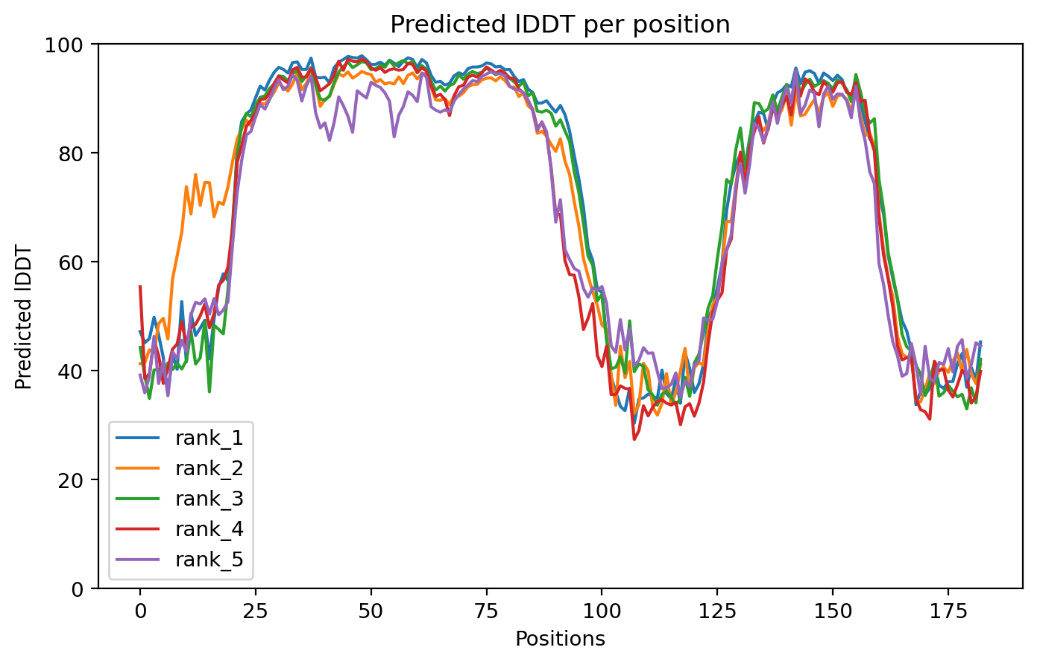

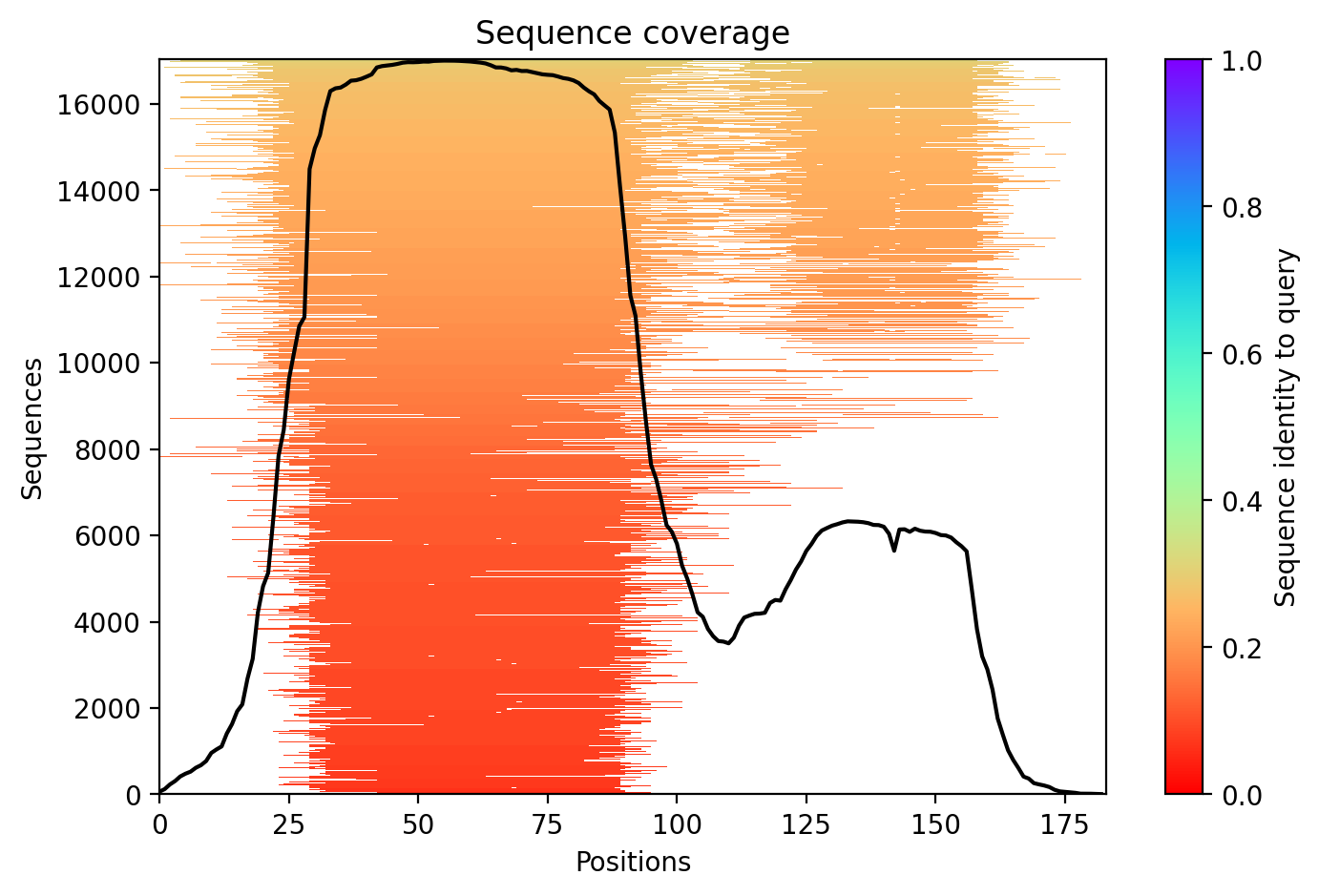

Supplement: Supplemental figures — Figures S1 and S2. [file msphere.00872-25-s0001.docx]
